# A *Dystrophin* Exon-52 Deleted Miniature Pig Model of Duchenne Muscular Dystrophy and Evaluation of Exon Skipping

**DOI:** 10.3390/ijms222313065

**Published:** 2021-12-02

**Authors:** Yusuke Echigoya, Nhu Trieu, William Duddy, Hong M. Moulton, HaiFang Yin, Terence A. Partridge, Eric P. Hoffman, Joe N. Kornegay, Frank A. Rohret, Christopher S. Rogers, Toshifumi Yokota

**Affiliations:** 1 Department of Medical Genetics, Faculty of Medicine and Dentistry, University of Alberta, Edmonton, AB T6G 2H7, Canada; echigoya.yusuke@nihon-u.ac.jp (Y.E.); ntrieu@unb.ca (N.T.); 2 Laboratory of Biomedical Science, Department of Veterinary Medicine, College of Bioresource Sciences, Nihon University, Kanagawa 252-0880, Japan; 3 Microscopy & Microanalysis Facility, University of New Brunswick, Fredericton, NB E3A 5A3, Canada; 4 Northern Ireland Centre for Stratified Medicine, Altnagelvin Hospital Campus, Ulster University, Londonderry BT47 6SB, UK; w.duddy@ulster.ac.uk; 5 Biomedical Sciences, College of Veterinary Medicine, Oregon State University, Corvallis, OR 97331, USA; hong.moulton@oregonstate.edu; 6 Department of Cell Biology, School of Medical Technology, Tianjin Medical University, Qixiangtai Road, Heping District, Tianjin 300070, China; haifangyin@tmu.edu.cn; 7 Research Center for Genetic Medicine, Children’s National Medical Center, Department of Integrative Systems Biology, George Washington University School of Medicine, Washington, DC 20010, USA; tpartridge@childrensnational.org; 8 School of Pharmacy and Pharmaceutical Sciences, Binghamton University, State University of New York, Binghamton, NY 13902, USA; ericphoffman@gmail.com; 9 College of Veterinary Medicine and Biomedical Sciences, Texas A&M University, College Station, TX 77843, USA; joe_kornegay@med.unc.edu; 10 Precigen Exemplar, 2656 Crosspark Rd. STE 100, Coralville, IA 52241, USA; frank.rohret@exemplargenetics.com

**Keywords:** DMD, dystrophin, pig model, exon skipping, antisense oligonucleotide, morpholino, large animal model

## Abstract

Duchenne muscular dystrophy (DMD) is a lethal X-linked recessive disorder caused by mutations in the *DMD* gene and the subsequent lack of dystrophin protein. Recently, phosphorodiamidate morpholino oligomer (PMO)-antisense oligonucleotides (ASOs) targeting exon 51 or 53 to reestablish the *DMD* reading frame have received regulatory approval as commercially available drugs. However, their applicability and efficacy remain limited to particular patients. Large animal models and exon skipping evaluation are essential to facilitate ASO development together with a deeper understanding of dystrophinopathies. Using recombinant adeno-associated virus-mediated gene targeting and somatic cell nuclear transfer, we generated a Yucatan miniature pig model of DMD with an exon 52 deletion mutation equivalent to one of the most common mutations seen in patients. Exon 52-deleted mRNA expression and dystrophin deficiency were confirmed in the skeletal and cardiac muscles of DMD pigs. Accordingly, dystrophin-associated proteins failed to be recruited to the sarcolemma. The DMD pigs manifested early disease onset with severe bodywide skeletal muscle degeneration and with poor growth accompanied by a physical abnormality, but with no obvious cardiac phenotype. We also demonstrated that in primary DMD pig skeletal muscle cells, the genetically engineered exon-52 deleted pig *DMD* gene enables the evaluation of exon 51 or 53 skipping with PMO and its advanced technology, peptide-conjugated PMO. The results show that the DMD pigs developed here can be an appropriate large animal model for evaluating in vivo exon skipping efficacy.

## 1. Introduction

Duchenne muscular dystrophy (DMD) is an X chromosome-linked recessive neuromuscular disorder caused by mutations in the *DMD* gene and the subsequent lack of dystrophin protein [1]. The incidence has been reported to be in the range of 10.7 to 27.8 cases per 100,000 live male births [2]. One of the most common mutation types is a deletion of entire exon(s) in the *DMD* gene, accounting for approximately 68% of the patients’ mutations [3]. The mutations lead to the *DMD* reading frame disruption referred to as out-of-frame and generate premature stop codons. Dystrophinopathy is characterized by progressive weakness and degeneration in bodywide skeletal muscles [4]. Patients are initially recognized due to their abnormal gait and lose ambulation by 10–13 years [5], accompanied by high serum creatine kinase levels [6]. With improved respiratory support, cardiac involvement is currently a leading cause of premature death in the 20 s to 40 s in affected individuals [7,8].

Dystrophic animal models have promoted understanding of DMD pathology, leading to the development of various candidate therapies aimed at restoring the expression of dystrophin protein or correcting physiological pathways involved in the pathogenesis [9,10]. Of these, currently, a most promising therapeutic approach is exon skipping using short, synthetic antisense oligonucleotides (ASOs) to restore the reading frame of *DMD* mRNA, thus generating, functional though truncated, dystrophin protein. In the past five years, four phosphorodiamidate morpholino oligomers (PMOs) have been conditionally approved by the U.S. Food and Drug Administration (FDA), including eteplirsen for exon 51 skipping [11], golodirsen [12], and viltolarsen [13] for exon 53 skipping, and casimersen for exon 45 skipping [14]. Encouragingly, an applied technology has already emerged using cell-penetrating peptide (CPP) conjugations to PMOs, enabling PMOs to be taken more efficiently into skeletal and cardiac muscle cells and to skip exon(s) of a target transcript [15,16].

Dystrophic mice and dogs have played significant roles in demonstrating the proof-of-concept of ASO-mediated exon skipping [17,18,19,20,21]; however, the murine and canine models have some limitations regarding their physical and genetic resemblance to patients [22]. First, the *mdx* mouse has a milder phenotype, raising a question as to whether findings will translate to patients. Second, their mutations, such as the point mutation in exon 23 in *mdx* mice are not commonly seen in DMD patients. Lastly, murine and canine anatomy and physiology are distant from humans, limiting interpretation and extrapolation of treatment effects from animal to human. Although the wide variety of currently available animal models may partially compensate for these drawbacks [23], a more suitable animal model, particularly a large animal model, is most desirable to overcome them.

As such a model, pigs, particularly miniature pigs, are scientifically accessible and socially more acceptable than canine models. In many respects, the pig is more similar to the human than most other models, potentially helping to fill the substantial gap between currently available animal models and patients. Also, they are more amenable to the cloning technology of somatic cell nuclear transfer (SCNT) to obtain genetically identical piglets, as we previously demonstrated [24]. In contrast, SCNT in dogs has been reported to suffer from poor efficiency [25]. This unique property enables the production of various pig models of human diseases combined with genetic engineering technologies such as gene targeting [26]. These advantages provide an opportunity to introduce deletion mutations in “hotspot” regions as seen in patients [27], increasing the applicability of transgenic pigs to preclinical testing. Encouragingly, the development of such cloned pigs with the genetically engineered mutant *DMD* gene has been progressing [28,29,30,31]. However, further development and characterization of DMD pig models are essential to reveal the value of their applicability to preclinical research and better understand their pathogenesis.

In this study, we used a combination method of recombinant adeno-associated virus (rAAV)-mediated gene targeting and SCNT to generate a tailored *DMD* mutant pig [24]. Consequently, we developed a Yucatan miniature pig model of DMD with an exon 52 deletion mutation that mimics one of the most common mutations in patients [27]. These resultant *DMD* exon 52-deleted pigs (DMD*^ex52del^* pigs) were molecularly and histologically characterized. Also, through in vitro tests with the primary DMD*^ex52del^* pig skeletal muscle cells, the present study reports the potential of DMD*^ex52del^* pigs for the development of CPP-conjugated PMO-mediated exon skipping.

## 2. Results

### 2.1. Generation of a Miniature Pig Model of Duchenne Muscular Dystrophy

**Figure 1:**
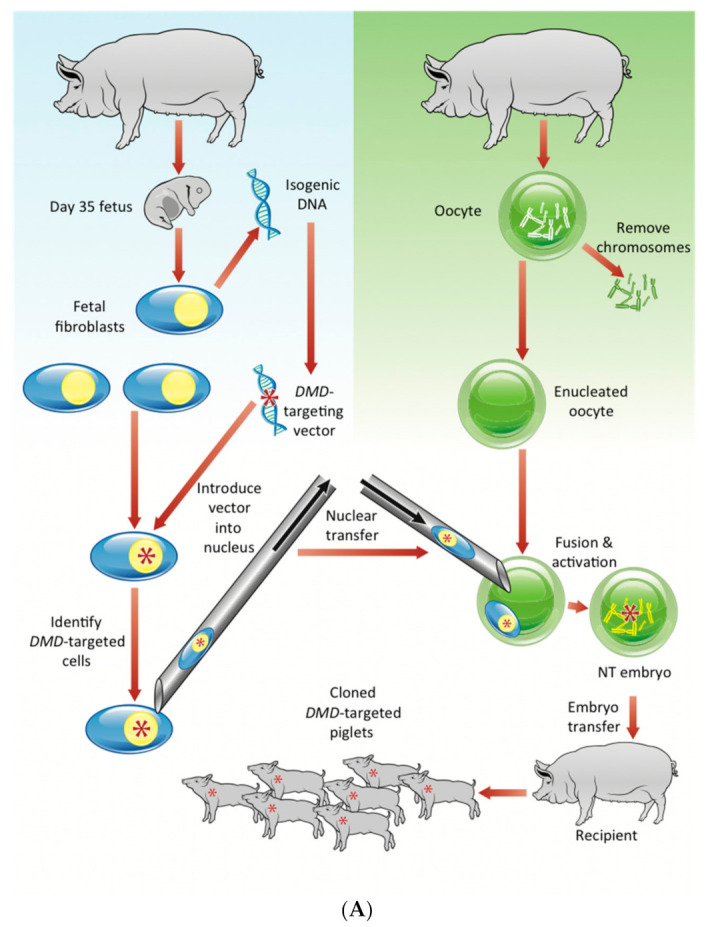

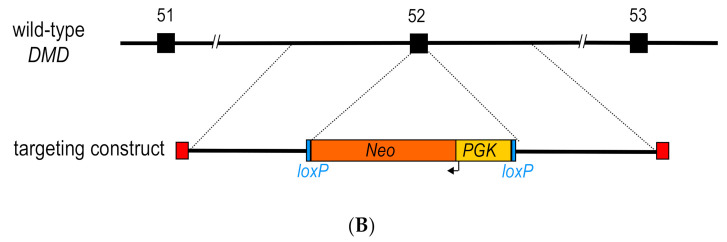
Overview of generation of the clonal miniature pigs with a *DMD* exon-52 deletion mutation. (**A**) Schematic of the combination method of the rAAV-mediated gene targeting and somatic cell nuclear transfer for producing the cloned *DMD*-targeted male piglets. Fibroblast cells derived from male pigs at the age of 35-days were obtained and infected with the rAAV packaging *DMD^ex52del^* targeted vector identified by a red asterisk. The *DMD*-targeted male pig fibroblast nucleus was transferred into enucleated oocytes, followed by fusion and activation in cells. The nuclear transfer (NT) embryos were transferred into the uterus of a recipient pig. After a 114 day gestation period, *DMD*-targeted piglets were produced. (**B**) Porcine *DMD* exon 52 was replaced with a neomycin resistance cassette (*Neo^R^*) (orange) driven by a phosphoglycerate kinase (PGK) promoter (yellow) and flanked by loxP sites (blue). Exons 51–53 of porcine *DMD* are depicted in black boxes. The rAAV inverted terminal repeats (ITRs) are in red. Each homology arm is ~1.4 kb in length. The figure is not to scale.

A Yucatan miniature pig model of DMD was generated by homologous recombination via rAAV–mediated gene targeting that creates an exon 52 deletion in the pig *DMD* gene and SCNT (Figure 1A) [24]. Male pig fetal fibroblasts were infected with rAAV carrying a targeting construct designed to replace the endogenous *DMD* exon 52 with a neomycin resistance cassette (*Neo^R^*) driven by the phosphoglycerate kinase (PGK) promoter, flanked by loxP sites (Figure 1B).

**Figure 2:**
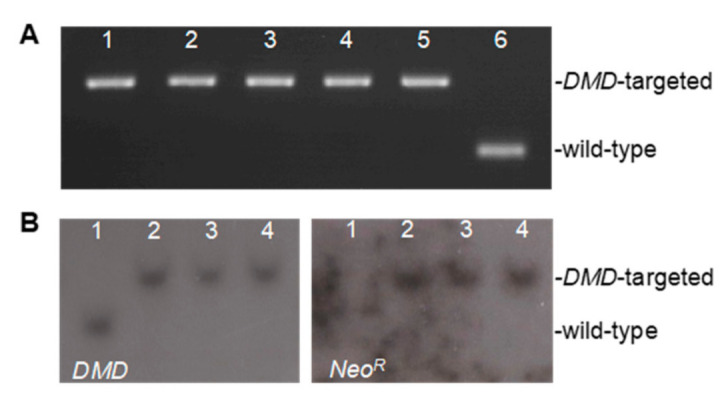

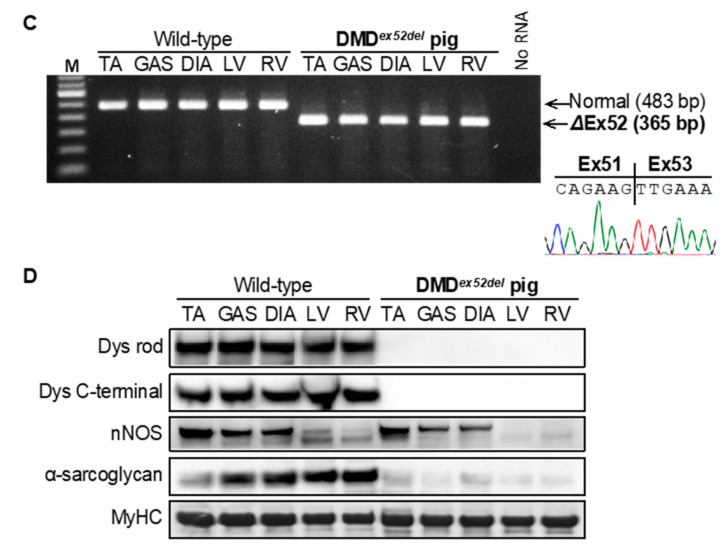
Molecular characterization of the *DMD* exon 52-deleted miniature pig model. (**A**) Genotyping PCR using umbilical cord tissue lysates confirmed the exon 52 deletion in the pig *DMD* gene. Lanes 1–5 represent PCR amplicons of the *DMD* region of DMD*^ex52del^* pigs. Lane 6 is a lysate from a wild-type pig. (**B**) Representative genomic Southern blot of 3 DMD*^ex52del^* pigs. (Left) MfeI-digested genomic DNA was hybridized with a probe that detects porcine *DMD* downstream of the targeting vector boundary. The *DMD*-targeted allele produced an approximately 6.3 kb band, and the wild-type band is 4.7 kb. (Right) The same DNA was hybridized with a probe that detects the *Neo^R^*, yielding only the targeted 6.3 kb band. Lane 1: wild-type; Lanes 2–4: individual DMD*^ex52del^* male pigs. (**C**) RT-PCR revealed exon 52-deletion in *DMD* mRNA in the skeletal and cardiac muscles. TA, tibialis anterior; GAS, gastrocnemius; DIA, diaphragm; LV, left ventricular free wall; RV, right ventricular free wall. (**D**) Lack of dystrophin and reduction in its associated protein levels in skeletal and cardiac muscles as represented by Western blotting. Dystrophin (Dys) rod domain and Dys C-terminal were detected by monoclonal antibodies, NCL-DYS1 and NCL-DYS2, respectively. nNOS, Neuronal nitric oxide synthase. MyHC, myosin heavy chain stained by Coomassie Brilliant Blue in a post-transferred gel.

The resulting *DMD* exon 52-deleted fibroblasts were used as nuclear donors for SCNT. We performed the SCNT with genetically engineered male fibroblasts, generating a total of seven affected males (Table S1). The genotype of each pig produced was confirmed by PCR using primers (Table S2) and Southern blotting using probes that hybridize the *DMD* region or *Neo^R^* sequence (Figure 2A,B, respectively). Of these seven piglets, five died within a week, and two survived up to 6.5 and 7 months of age, respectively. Expression of exon 52-deleted mRNA in skeletal and cardiac muscles was shown by RT-PCR and the boundary of exons 51 and 53 was confirmed by direct sequencing (Figure 2C), validating the out-of-frame mRNA in the affected pigs. Dystrophin was absent in skeletal and cardiac muscles as shown by Western blotting with antibodies against the dystrophin rod and C-terminal domains (Figure 2D). Western blotting also revealed reduced expression levels of dystrophin-associated proteins (DAPs), neuronal nitric oxide synthase (nNOS) and α-sarcoglycan.

### 2.2. Dystrophin Deficiency Causes Failed Recruitment of DAPs to the Sarcolemma of the Transgenic Pig Model

**Figure 3:**
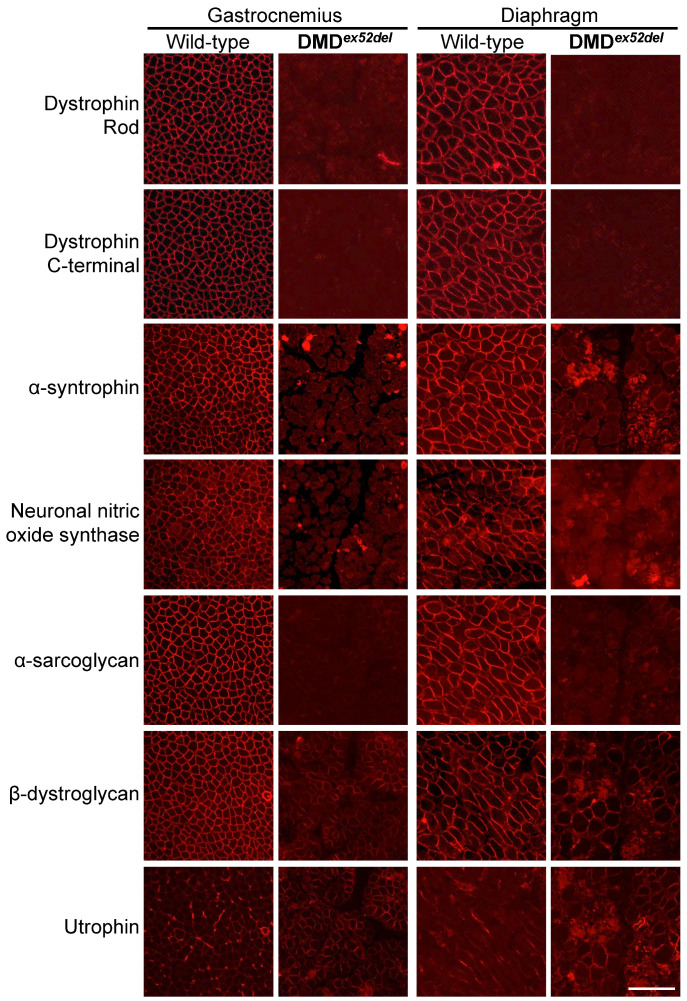
Reduction in dystrophin and dystrophin-associated proteins in serial sections of DMD*^ex52del^* pig skeletal muscles. Dystrophin localization was analyzed using antibodies against the rod domain (ab85302, Abcam) or C-terminal (ab15277, Abcam). Other antibodies used here are shown in Table S3. Representative images in the quadriceps and diaphragm muscles of the dystrophic pig (ID 9290) at the age of 5 days are shown. Bar, 100 μm.

Immunohistochemistry with serial frozen sections of skeletal muscles of the DMD*^ex52del^* pig model revealed the lack of dystrophin in the muscle membrane, accompanied by reduced expression of DAPs (Figure 3). The DAPs such as α-syntrophin, nNOS, α-sarcoglycan, and β-dystroglycan were not recruited to the muscle membrane. The signal intensity of sarcolemmal utrophin, a homolog of dystrophin, was slightly increased in the affected pig skeletal muscles compared to wild-type pigs, as seen in patients [32] and other murine [33] and canine [34] models.

### 2.3. Severe Pathology in DMD^ex52del^ Pig Muscles

**Figure 4:**
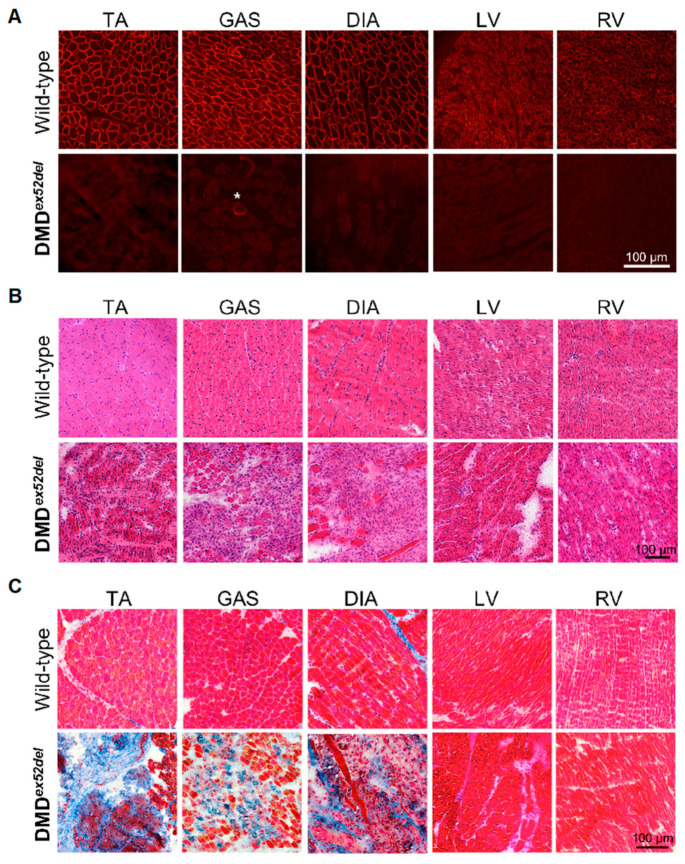
Muscle degeneration in the dystrophin-deficient DMD*^ex52del^* pigs. (**A**) Dystrophin deficiency in skeletal and cardiac muscles was confirmed by immunohistochemistry with anti-dystrophin rod domain antibody (NCL-DYS1). An asterisk identifies a dystrophin positive-revertant fibre. Muscle degeneration was assessed in (**B**) H&E staining and (**C**) Masson’s trichrome staining. Blue indicates collagenous connective tissues. Representative images of an affected male piglet (ID 9290) at the age of 5 days are shown.

The absence of dystrophin expression was confirmed in the DMD*^ex52del^* pig model’s skeletal and cardiac muscles using immunohistochemistry (Figure 4A). Dystrophin-positive revertant fibres were sporadically observed as single fibres on the dystrophin-negative muscle background, as shown in Figure 4A. A cluster as seen in mouse models was not found through observation [35]. Accompanying the lack of dystrophin protein, severe degeneration was seen in skeletal muscles in the DMD piglets from 5 days of age with haematoxylin and eosin (H&E) staining and Masson’s trichrome staining (Figure 4B,C). Centrally nucleated fibres were observed in the skeletal muscles in H&E staining. Necrotic fibres and fibrosis were confirmed in the skeletal muscles of the tibialis anterior muscle, gastrocnemius muscle, and diaphragm muscle on Masson’s trichrome staining. Unlike the skeletal muscles, the left and right ventricles of the heart did not show noticeable histological changes.

### 2.4. DMD^ex52del^ Pigs Exhibit Muscle Weakness and Poor Growth Rate

**Figure 5:**
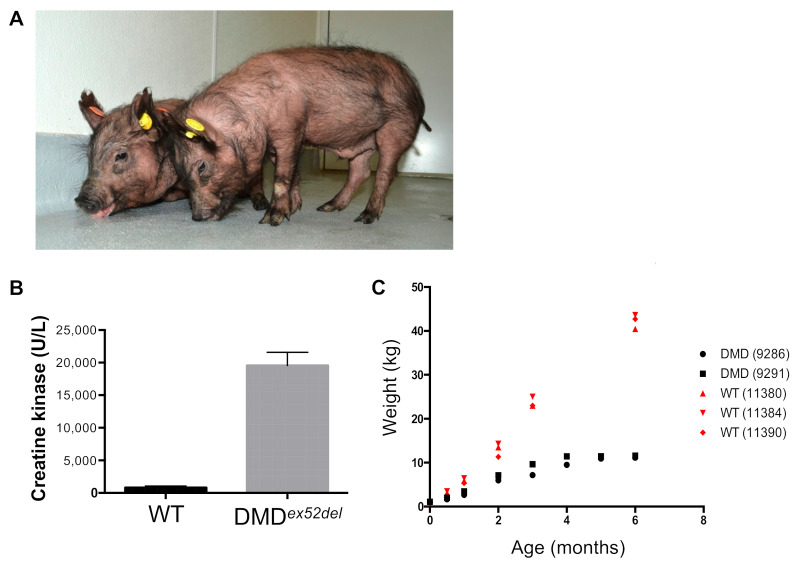
DMD*^ex52del^* pigs display dystrophic phenotypes in clinical assessment. (**A**) Physical abnormality in male DMD^e*x52del*^ pigs at 15 weeks of age (IDs 9286 and 9291) with postural instability and associated forward positioning of the pelvic limbs. (**B**) Serum creatine kinase measurements from wild-type (*n* = 3) and DMD pigs (*n* = 4) on the day of birth. Error bars indicate SEM. (**C**) The poor growth rate of DMD pigs compared to age-matched wild-type (WT) pigs.

On phenotypic assessment, we observed progressive muscle weakness in DMD*^ex52del^* pigs (IDs 9286 and 9291) with age (Figure 5A), essentially identical to dog models of DMD [19,22,36]. The DMD*^ex52del^* pigs demonstrated less activity and dystrophic phenotypes such as an enlarged tongue (macroglossia), postural instability, and associated forward positioning of the pelvic limbs as seen in another large animal model, the GRMD dog [36]. Serum creatinine kinase was severely elevated to an average 19,581 U/L, standard error of the mean (SEM) ± 2021 (to convert U/L to μkat/L, multiply by 0.0167) in DMD*^ex52del^* pigs within 24 h after birth compared to that in wild-type pigs (an average 816 U/L, SEM ± 267) (Figure 5B), consistent with what is seen in newborn patients and other animal models [6,37]. Growth of dystrophic pigs was remarkably impaired from 2 months of age compared to age-matched wild-type miniature pigs (Figure 5C). The affected pigs were euthanized around seven months of age due to their diminished body condition and weight loss.

### 2.5. Design of Pig Antisense Oligonucleotide Sequences and Prediction of Exon Skipping Efficiency

For testing exon skipping feasibility in the mutant pig *DMD* gene, we first computationally designed a total of 204 and 183 30-mer ASO sequences for use with PMO chemistry, to skip pig *DMD* exons 51 and 53, respectively (Tables S4 and S5). Pig ASOs were designed based on a prediction of exon-skipping efficiency for each possible target site across the entire sequence of each target exon. The prediction was facilitated by a computational tool we previously developed to design ASOs to skip human *DMD* exons [38]. The in silico prediction revealed that the upstream region of both pig *DMD* exons 51 and 53 could be optimal for skipping efficacy (Figure S1A,B). The effective and ineffective regions computationally calculated for the pig *DMD* exons were similar to those for humans (Figure S1C,D), suggesting the potential utility of our algorithms to design effective ASO sequences for the animal gene. For empirical tests, we selected five ASOs for each exon based on the ranking with the predicted skipping efficiency values and on referring to the previous achievement of the test in the patient *DMD* gene (Table 1). Namely, for ASOs tested to skip exon 51, pEx51_Ac0 and pEx51_Ac48 were the analogues of the ASOs that were the most and second-most effective, respectively, at skipping human *DMD* exon 51 in vitro and in vivo [39]. pEx51_Ac5 had the highest predicted value (89.1%) in the present calculation. pEteplirsen_Ac65 and pDrisapersen_Ac67 were the pig version PMO sequences of the FDA-approved PMO, eteplirsen [40] and clinically trialed 20-mer 2′-O-methyl phosphorothioate oligonucleotide, drisapersen, respectively [41]. Pig *DMD* exon 53 skipping ASOs were selected from among the target sites having the top thirty of the predicted skipping values. Of them, pEx53_Ac26, which had the tenth-highest ranking for pig target sites, was the analogue sequence of the human site that had the greatest efficiency at skipping exon 53 in the DMD patient-derived skeletal muscle cells [38]. pEx53_Ac9 was a 30-mer sequence with the top-ranked skipping value in the prediction of 88.8%. All ASOs were synthesized as PMOs (Gene Tools).

### 2.6. PMO-Mediated Exon Skipping Is Feasible in DMD^ex52del^ Pig Skeletal Muscle Cells In Vitro

**Figure 6:**
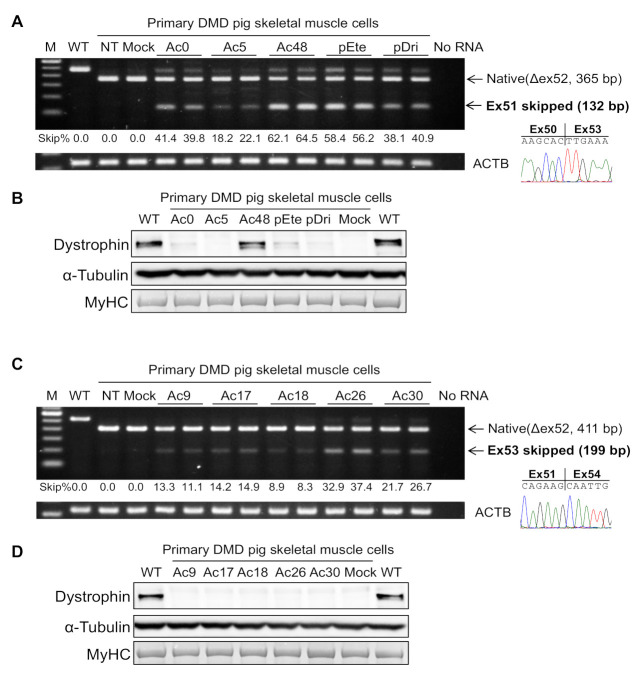
The feasibility of *DMD* exon 51 or 53 skipping in the DMD*^ex52del^* pig skeletal muscle cells in vitro. Exon 51 skipping efficiency (**A**) and expression levels of rescued dystrophin protein (**B**) were analyzed in primary DMD pig muscle cells treated with 10 μM PMOs and 6 μM transfection agent, Endo-Porter (the culture condition is shown in Figure S2A). The efficiency of exon 53 skipping using pig PMOs was also tested in primary DMD pig cells (**C**), but no dystrophin was detected on Western blotting (**D**). pEte, the pig version PMO of FDA-approved eteplirsen; pDri, the pig version PMO of drisapersen previously tested in a clinical trial as 2′-O-methyl phosphorothioate oligonucleotides. WT, wild-type; NT, non-treated; Mock, 31-mer negative control PMO. Alpha-tubulin and MyHC were used as a loading control and marker of myogenic differentiation, respectively. Representative results from three independent experiments using two DMD pig cell lines derived from pigs with IDs 9594 and 9595 are shown.

To evaluate exon skipping PMO potency in pig muscle cells, we generated primary skeletal muscle cells from wild-type and DMD*^ex52del^* miniature pigs (IDs 9594 and 9595). The maturation of the differentiated pig skeletal muscle cells appropriate for testing exon skipping was confirmed by the expression of *DMD* mRNA produced at the late stage of myotube formation during myogenesis (Figure 6) [39].

PMOs (10 μM) were transfected to the myotubes differentiated from the primary DMD pig skeletal muscle cells at the fourth or fifth passage (Figure S2A). All PMOs tested succeeded in skipping exon 51 or 53 in the DMD pig skeletal muscle cells as represented by RT-PCR (Figure 6 and Figure S3). In the in vitro test of exon 51 skipping, pEx51_Ac48 PMO showed the greatest efficiency, reaching up to 64%, followed by pEteplirsen_Ac65 PMO (Figure 6A and Figure S3A). Interestingly, 20-mer pDrisapersen_Ac67 PMO at 10 μM induced up to 40% exon skipping efficiency in the DMD pig muscle cells, whereas 10 μM human PMO composed of drisapersen sequence had been reported to have no practical ability to skip exon 51 in a DMD patient muscle cell line with the same exon 52 deletion mutation using the identical method to the present study [39]. The boundary composed of exons 50 and 53 in the exon 51-skipped transcripts was confirmed by direct sequencing, indicating the induction of in-frame mRNA following the treatment of exon 51 skipping PMOs. Accordingly, the rescued dystrophin protein was observed on Western blotting in the DMD pig cells treated with PMOs, with a particularly strong signal for pEx51_Ac48 PMO (Figure 6B). Five PMOs for exon 53 skipping also successfully induced in-frame transcripts at the exon 51-54 junction in primary skeletal muscle cells following the treatment (Figure 6C and Figure S3B). The highest efficiency for exon 53 skipping was 37% for pEx53_Ac26 PMO. Dystrophin rescue was not detected by Western blotting in pig cells treated with exon 53 skipping PMOs due to their lower exon skipping efficiencies, consistent with exon 51 skipping result using pEx51_Ac5 or pDrisapersen PMOs that showed no apparent rescued protein bands.

### 2.7. Peptide Conjugation to PMOs Potentiates Exon Skipping Efficiency in DMD^ex52del^ Pig Skeletal Muscle Cells

Cell-penetrating peptide, CPP, conjugation to PMOs is reported to increase efficacy at skipping target exons and overcome PMO chemistry limitations such as relatively poor delivery to muscle cells [42,43]. To examine if the pig model developed here is amenable to such a CPP technology, we tested the in vitro efficacy of a CPP-, P7-conjugated PMOs (P7-PMOs: Figure S2C) [44] with pEx51_Ac48 and pEx53_Ac26 sequences, confirming these PMOs were more effective at skipping target exons, in primary DMD*^ex52del^* pig cells. Primary DMD*^ex52del^* pig skeletal muscle cells were transfected with P7-conjugated PMOs or unmodified PMO with or without a peptide-based transfection reagent, Endo-Porter (Gene Tools) in the range of 125 nM to 5 μM, according to the culture schedule as shown in Figure S2C.

**Figure 7:**
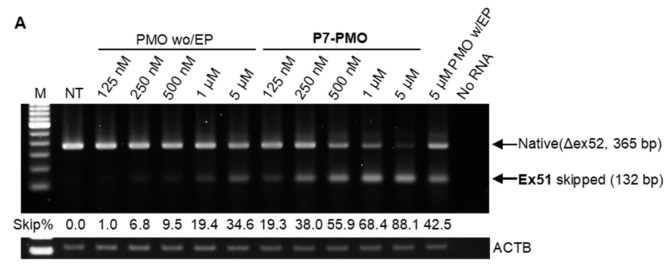

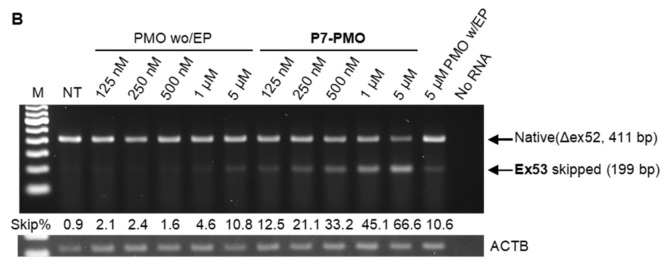
A cell-penetrating peptide (P7)-conjugation potentiates the in vitro efficacy of morpholinos (PMOs) at skipping exon 51 or 53 in the primary DMD*^ex52del^* pig muscle cells as represented by RT-PCR. (**A**) Exon 51 skipping efficiency comparison between unmodified PMO with or without a transfection agent, Endo-Porter (EP) at 6 μM, and P7-peptide-conjugated PMO that have the Ac48 sequence. (**B**) Exon 53 skipping induced by Ac26-PMO with or without EP or with P7-conjugation. M, 100 bp marker; NT, non-treated.

On RT-PCR, whereas unmodified PMO tested at the highest concentration of 5 μM without Endo-Porter induced up to 34% exon 51 skipping efficiency, similar effectiveness was achieved with P7-PMO at the lower quantity of 250 nM (Figure 7A). The highest efficiency for exon 51 skipping was 88% for 5 μM P7-PMO. Consistent with the exon 51 skipping result, exon 53 skipping P7-PMO tested at the lowest concentration (125 nM) showed up to 13% efficiency (Figure 7B). Such a level was found with unmodified PMO at the highest concentration of 5 μM. In P7-PMO-mediated exon 53 skipping, the highest concentration (5 μM) induced up to 66% skipping efficiency. Together, P7-PMOs were approximately 20- and 40-times more efficient in exon 51 and 53 skipping, respectively, than unmodified PMOs concentration-wise, when tested in primary DMD*^ex52del^* pig skeletal muscle cells. Interestingly, in both exon 51 and 53 skipping, we found that the Endo-Porter peptide reagent, unlike P7-conjugation, did not potentiate in vitro skipping ability of unmodified PMO at 5 μM. Direct sequencing confirmed the junction of exons 50 and 53 or exons 51 and 54 of the exon-skipped transcripts.

## 3. Discussion

With preclinical studies using rodent and canine models, numerous efforts have been made to develop therapeutic approaches for DMD such as small molecules, stop codon readthrough, gene replacement, and exon skipping [10]. Dystrophic mouse models, notably a series of *mdx* models, are valuable tools in the proof-of-concept stages of drug candidates because the strains have been established and well characterized. Novel dystrophic models with mice and rats are also further along in development utilizing their facility for genetic manipulation [45,46,47]. On the other hand, canine models of GRMD and CXMD_J_ have played pivotal roles in DMD research, as they display many of the clinical manifestations seen in patients. While very useful, their limitations in physical similarity, severity, or mutations have hampered moving the therapeutic approaches to the clinic.

Given a need for an appropriate model system to facilitate the discovery of drugs, in the present study, using SCNT following rAAV-mediated gene targeting, we developed a *DMD* exon 52-deleted miniature pig model that shows a lack of dystrophin protein bodywide and a severe phenotype. As we previously reported [48], the cloning technology of SCNT is an effective method for generating transgenic piglets compared to other methods with porcine embryonic stem cells and induced pluripotent stem cells, which have some limitations such as cell isolation difficulty, incomplete reprogramming, and a lower rate of embryonic development in pigs [49,50,51]. The present study indicates that the SCNT combined with gene targeting by the rAAV vector used here can facilitate generation of DMD models with pigs harbouring genetic mutations of interest, together with other genetic engineering technologies such as genome editing [28,29,52,53].

The molecular and histological evaluation in our study confirmed that our clonal DMD miniature pigs with the exon 52 deletion, DMD*^ex52del^* pigs, have severe phenotypes with bodywide skeletal muscle degeneration. The model also manifested no dystrophin protein expression in skeletal muscle, high serum creatine kinase levels, and progressive disease status as seen in poor growth rate. Disrupted DAP assembly and revertant fibre expression further demonstrate that the DMD*^ex52del^* pig muscles manifest dystrophic pathology features, as observed in the established murine and canine models [52,54]. The increased levels of utrophin in the sarcolemma indicate that the DMD*^ex52del^* pigs may have a similar compensatory mechanism for the dystrophin deficiency to that found in other animal models [33,34].

Another distinctive feature is that the dystrophin-deficient cardiac muscle of our DMD*^ex52del^* pig model showed the disrupted expression levels of DAPs but no obvious histopathological abnormalities. Since cardiomyopathy due to the absence of dystrophin is more manifested with advancing age in patients [53] and canine models [55,56], pig models may also display histological and functional abnormalities in the heart later during life. Detailed cardiac assessment of the DMD pigs will be a challenge due to their shorter lifespan.

A report of similar severity in another cloned porcine DMD model with the same exon 52-deletion but a different genetic background also substantiates the value of the tailored DMD*^ex52del^* pig model as a severe dystrophic model for a better understanding of DMD pathology [28]. Interestingly, consistent with our results, their DMD*^ex52del^* pigs have exhibited severe degeneration in skeletal but not in cardiac muscle. They also manifested a reduced growth rate as seen in the present study, surviving only up to 3 months of age; shorter than the 7 months we noted with our DMD*^ex52del^*. A DMD miniature pig model with a Chinese Diannan breed has also been reported with the CRISPR/Cas9 system used for *DMD* exon 27-targeting-knockout, showing a short lifespan of 52 days [29]. In the present study, 5 out of 7 affected male piglets died or were euthanized within a week. Such early neonatal death is reported as a fulminant neonatal form in some puppies of the GRMD dog model [36], though the pathogenic mechanism remains unclear. Associated with the fulminant case, it also needs to be investigated how epigenetic dysregulation in genetically modified pig cells influences phenotypic instability [57,58]. The severe pathology observed in the porcine models reported so far may provide insight into the mechanism of progressive dystrophic changes in skeletal muscles. In addition, phenotypic variation between strains or littermates of DMD pigs needs to be characterized for pig models to be used in the preclinical field.

While the progressive and severe signs in DMD*^ex52del^* pigs partially resemble DMD patients, the rapidity and severity of their disease may raise a concern when distributing them as a tool for translational research; i.e., it could mask the therapeutic effects of drugs tested. To overcome such a limitation, Nagashima’s group, using chimeric embryos of normal and genetically modified blastomeres prepared by SCNT, has previously reported generating a DMD*^ex52del^* pig model showing a milder dystrophic phenotype [30]. Given the severity of the cloned models, the establishment and maintenance of the generated mutant strains represent a significant challenge in developing the DMD pig model. As described previously, male DMD pigs, including our model, did not reach the age or keep the health condition to allow for breeding, indicating that affected piglets need to be prepared by SCNT when one needs them. Efforts to generate a carrier of female pigs with heterozygous *DMD* mutation in an X-chromosome allele (*DMD*^+/−^) have been made with genome editing of germinal oocytes [59]. Encouragingly, Wang and Lei’s group and Kupatt’s group have reported heterozygous female *DMD*^+/−^ pigs that enable the production of affected male *DMD*^Y/−^ offspring [31,60]. We also succeeded in generating female miniature pig-derived fibroblast cell lines that harbour the *DMD*^+/−^ genotype composed of wild-type and exon 52-deleted alleles (data not shown), paving the way to generate the carrier miniature sow through SCNT as we previously demonstrated for a pig model of another genetic disease, cystic fibrosis [48]. As there is limited information regarding the phenotype of DMD carrier female pigs and non-cloned DMD*^ex52del^* male offspring, further characterization is needed to compare the phenotype of non-cloned DMD*^ex52del^* male offspring with clonal male founders produced using SCNT.

An advantage of the exon 52 deletion we created in pigs is that this mutation theoretically allows for evaluating exon 51 and exon 53 skipping to treat the highest proportion (19.6% and 15.3%, respectively) of patients having *DMD* out-of-frame deletion mutations [27]. For the same reason, a dystrophic mouse model with mouse *Dmd* exon 52 deletion, called an *mdx52* mouse, was generated [61]. The *mdx52* mice significantly contributed to establishing the proof-of-concept of exon 51 and 53 skipping therapies for which PMO-based ASO drugs have become commercially available [4,62]. While mouse models act as a hub for DMD research, DMD*^ex52del^* pigs, being closer to patients in terms of many physical traits, are expected to become a practical approach to bridge the gap between pre-clinical and clinical results. Here, we confirmed that the exon 52-deleted *DMD* gene is amenable to both PMO-meditated exon 51 and 53 skipping in vitro, correcting the reading frame in DMD*^ex52del^* pig-derived skeletal muscle cells. Thus, we demonstrated that the primary skeletal muscle cells of DMD*^ex52del^* pigs serve as a valuable tool for sensitive ASO screening before the validation using live animals. Towards in vivo testing, unanticipated splicing events involving exon(s) other than a target exon need to be investigated to make the exon-skipping results in the model more reliable.

Another important finding is that the ASO predictive tool that we have developed for human *DMD* [38] can be used to discover effective pig PMO sequences. Interestingly, the profiles of predicted skip efficacy across pig exons 51 and 53 resembled those of humans. The robustness of the pig ASO prediction was validated with the empirical assay of exon skipping in the primary DMD*^ex52del^* pig skeletal muscle cells. In the proof-of-concept stage, a time-consuming in vitro screening using animal cells is needed to identify ASOs to be used for in vivo testing in animal models. Our ASO predictive tool can help to find effective ASO sequences to skip exon(s) of interest by narrowing down the candidate ASOs from among the hundreds of potential target sites on a given exon [63], as shown in Tables S4 and S5. Although current procedures are effective, there is a need to improve the accuracy of predictive algorithms as more data become available on different lengths, chemistries, and sequence-specific safety of ASOs. An effective in silico tool can reduce the time spent finding effective ASOs and accelerate the pre-clinical process when used in combination with an appropriate animal and its cell models such as DMD*^ex52del^* pigs. Indeed, our combination method using in silico and in vitro screening identified candidate PMO sequences, particularly pEx51_Ac48 and pEx53_Ac26 sequences, to be used for in vivo testing in the DMD pigs.

The PMO chemistry is one of the optimal chemistries for clinical application for exon skipping, as evidenced by the recent regulatory approvals [4]; however, its limited efficacy has been raised as a challenge requiring improvement [40]. The conjugation of a CPP to PMOs has attracted attention as a solution because it increases exon skipping efficacy in both patient cells and animal models [15]. We have previously demonstrated the increased potency of CPP-conjugated PMOs in skeletal and cardiac muscles of the CXMD_J_ dog model [21]. Currently, various CPPs including the P7-peptide used here have been developed and tested, aiming at clinical use, which requires evaluations of efficacy and safety using appropriate in vitro and in vivo models. Indeed, an immortalized CXMD_J_ skeletal muscle cell model was recently developed for PPMO screening [43]. The present study also demonstrated that the DMD*^ex52del^* pig-derived skeletal muscle cells allow for the evaluation of PPMO efficacy in vitro; the P7 conjugation potentiates by up to 40-fold the ability of a given concentration of PMO to skip pig *DMD* exon 51 or 53 than is the case without the conjugation, as shown in Figure 7. This result is encouraging for in vivo testing of the PPMO-mediated exon skipping approach in DMD*^ex52del^* pigs.

In conclusion, using a combination method of rAAV-mediated gene targeting and SCNT [24], we developed a tailored *DMD* mutant miniature pig model with an exon 52 deletion mutation frequently seen in patients. Consequently, we successfully produced clonal DMD*^ex52del^* pigs that display progressive and severe signs accompanied by a lack of dystrophin protein and disruption of DAPs’ assembly in body-wide skeletal and heart muscles. Through in vitro testing using the primary DMD*^ex52del^* pig skeletal muscle cells, our findings indicate the potential for DMD*^ex52del^* pigs to serve as an in vivo tool for evaluating the efficacy and safety of exon skipping induced by ASOs, and in particular, the more recently developed CPP-conjugated PMOs. Although there are technical and financial challenges in developing large animal models, generating various pig models with *DMD* mutations of interest and establishing a steady offspring supply could provide an effective tool to help the development of therapies for DMD patients.

## 4. Materials and Methods

### 4.1. Animals

All animals used in the present study were developed and housed in the Public Health Service (PHS)-assured facilities of Exemplar Genetics (Sioux Center, IA, USA). Standards and recommendations outlined in the Guide for the Care and Use of Laboratory Animals (NRC, 2011) and the PHS Policy were applied to husbandry and animal care. The Institutional Animal Care and Use Committee of Exemplar Genetics approved all animal experiments. Exemplar Genetics is also AAALAC accredited. The pigs were euthanized by i.v. injection of pentobarbital sodium (150 mg/kg; Intervet/Merck Animal Health, Kenilworth, NJ, USA) [64] following American Veterinary Medical Association guidelines. Subsequently, skeletal and cardiac muscles were collected for histological and molecular analyses.

### 4.2. Fetal Fibroblasts

Porcine fetal fibroblasts were prepared from male Yucatan miniature pig fetuses at day 35 as previously described [24,65]. Briefly, fibroblasts were cultured at 39 °C in F10 media (Invitrogen, Carlsbad, CA, USA), containing 20% FCS and 30 μg/mL gentamicin. The sex of the fetuses was determined by PCR for the Y chromosome-specific *Sry* gene [66].

### 4.3. Targeting Vector Construction

The pig *DMD* targeting vector was essentially constructed according to previous studies [24,67]. Genomic DNA was extracted from the fetal fibroblasts prepared above (Qiagen, Germantown, MD, USA). A DNA region including *DMD* exon 52 and flanking introns was amplified using a high fidelity polymerase (Platinum Taq High Fidelity, Invitrogen). The PCR product was sub-cloned into pCR2.1-TOPO (Invitrogen) and sequenced. The resulting plasmid was used as a template for PCR amplification of the 5′ and 3′ homologous targeting arms sub-cloned sequentially into a plasmid containing a cassette of the phosphoglycerate kinase promoter and neomycin resistance cDNA. The construct was designed to replace the endogenous *DMD* exon 52 with the cassette (Figure 1B).

### 4.4. rAAV Vector Production

The targeting vector sequence with a 4.5 kb amplicon size described above was sub-cloned into the rAAV2 pro-viral plasmid, pFBAAV2-CMVP.NpA (obtained from University of Iowa Viral Vector Core Facility, Iowa City, IA, USA) and grown in Sure2 competent cells (Stratagene, La Jolla, CA, USA), as previously described [24]. The rAAV was produced with the help of the University of Iowa Viral Vector Core Facility.

### 4.5. Generation of DMD Exon 52-Taregetted Pig Fibroblasts

Male fetal fibroblasts were treated with the rAAV vector prepared above to remove *DMD* exon 52 from the pig genome via homologous recombination as previously described [24]. In brief, 24 h after seeding the primary fibroblasts (1.5 × 10^6^) that were cultured without passaging, the cells were infected with the rAAV having the *DMD* targeting construct. Twenty-four hours after the infection, the cells were trypsinized and transferred to 96-well collagen-coated plates (BD Biosciences, San Jose, CA, USA). In 48 h, G418 (100 μg/mL) was added to the individual wells to select the targeted cells. Ten days later, each well was trypsinized (60 μL trypsin, 0.5% EDTA) for the cell collection. *DMD* exon 52 deletion and replacement by the *Neo^R^* was confirmed through PCR screen and direct-sequencing.

### 4.6. Somatic Cell Nuclear Transfer and Embryo Transfer

To produce the affected male piglets, SCNT and embryo transfer to recipient gilts were performed by Trans Ova Genetics (Sioux Center, IA, USA) and Exemplar Genetics as previously described [24,68]. Briefly, metaphase II chromosomes and the polar body were aspirated from oocytes by a micropipette, and then the donor fibroblast was deposited into the enucleated oocytes. Following the fusion and activation, the reconstructed oocytes were transferred into synchronized postpubertal domestic gilts on the first day of standing estrus under general anesthesia with i.v. propofol (0.5–5 mg/kg) and the inhaled isoflurane (3–5% in oxygen via face mask). Recipient animals were checked for pregnancy by abdominal ultrasound after day 21 and throughout a 114 day gestation period.

### 4.7. Genotyping Assay and Southern Blotting

Cloned piglets were genotyped to confirm the mutation with *DMD* exon 52 deletion by PCR (Figure 2A). The deletion mutation in the genomic DNA was further validated by Southern blotting using a probe that detects the pig *DMD* downstream of the targeting boundary or *Neo^R^* sequence (Figure 2B) as previously described [24].

### 4.8. Creatine Kinase Levels

Blood samples were collected from pigs within 24 h after birth to test serum creatine kinase levels [69]. Samples were submitted to a commercial laboratory (Marshfield Labs) for the measurement.

### 4.9. Tissue Samples

Skeletal and cardiac muscle blocks from wild-type and affected pigs were prepared as previously described [70]. Muscle samples were obtained immediately after the euthanasia and snap-frozen in liquid nitrogen-cooled isopentane. The samples were stored at −80 °C before use.

### 4.10. Histological Analyses

Skeletal and cardiac muscles were sectioned at 7–10 μm for histological analyses. H&E staining was conducted as previously described [35]. Masson’s trichrome staining was performed according to the TREAT-NMD SOP MDC1A_M.1.2.003. For immunohistochemistry with serial sections of muscle tissues, the unfixed sections were incubated with antibodies listed in Table S1. The signals visualized through fluorescence-conjugated secondary IgG antibodies were evaluated at 200× magnification using a Nikon Eclipse TE2000-U microscope (Nikon, Mississauga, ON, Canada), as previously described [52].

### 4.11. RT-PCR

Total RNA from frozen tissue sections or cultured cells was extracted with TRIzol reagent (Invitrogen) [21,39]. RT-PCR was performed in a 25-μL mixture containing 280 ng RNA using the SuperScript III One-Step RT-PCR System with Platinum Taq DNA Polymerase (Invitrogen) and primers (Table S2) following the manufacturer’s instructions. The cycling conditions are as follows: 50 °C for 10 min; 94 °C for 2 min; 35–40 cycles at 94 °C for 15 s, 60 °C for 30 s, and 68 °C for 30 s; and 68 °C for 5 min. PCR products were electrophoresed on a 1.5% agarose gel and visualized by SYBR Safe DNA Gel Stain (Invitrogen). The images were pictured in the KODAK Image Station 4000 MM (Kodak, Rochester, NY, USA) or the ChemiDoc Touch imaging system (Bio-Rad, Mississauga, ON, Canada).

### 4.12. Western Blotting

Protein extraction from frozen muscle sections or cultured cells was performed as previously described [52,63]. In brief, 30–50 μg of protein of the muscle sections or cells were loaded onto a NuPAGE Novex 3–8% Tris-Acetate or 4–12% Bis-Tris Midi Gel (Invitrogen) and separated by sodium dodecyl sulfate-polyacrylamide gel electrophoresis at 150 V for 75 min. The proteins were transferred onto an Immobilon PVDF membrane (Millipore) by semidry blotting at 20 V of constant voltage for 70 min. The membrane was blocked with phosphate-buffered saline containing 0.05% Tween 20, 0.1% casein and 0.1% gelatin, or 3% ECL blocking agent (GE Healthcare, Mississauga, ON, Canada). Blots were reacted with primary antibodies listed in Table S3 at 4 °C overnight, followed by the incubation with horseradish peroxidase-conjugated secondary IgG antibodies appropriate to react with the primary antibodies. Following the incubation with a chemiluminescent reagent (ECL Select, GE Healthcare), images of the blots were captured in the KODAK Image Station 4000 MM or the ChemiDoc Touch imaging system. Myosin heavy chain (MyHC) stained by Coomassie Brilliant Blue in post-transferred gels served as a loading control for individual muscle samples. In cell experiments, because of heterogeneous populations of primary cells, i.e., varying proportions of myogenic cells between individual primary pig cells established, primary healthy pig skeletal muscle cells were used as a qualitative index.

### 4.13. Design and Synthesis of Antisense Oligonucleotides

All possible ASO sequences 30-mer in length were considered for skipping exon 51 or 53 in the pig *DMD* gene (Tables S4 and S5, respectively). Exon skipping efficiencies of the designed ASO sequences were quantitatively predicted using the previously developed computational tool (Figure S1) [38]. The predicted values were evaluated to select ASOs to be used for the empirical test. ASO sequences experimentally tested were synthesized with the PMO chemistry by Gene Tools. PMOs with Ac48 and Ac26 to skip pig *DMD* exon 51 and 53, respectively, were conjugated with P7-peptide by H.M.M. (Oregon State University) [44].

### 4.14. Primary Skeletal Muscle Cells of DMD Pigs

Skeletal muscle tissues from two wild-types (IDs 14425 and 14426) or transgenic DMD pigs (IDs 9584 and 9585) were washed and collected in a sterilized PBS(-) immediately after euthanizing them. Ten grams of skeletal muscles, of which the damaged areas and connective tissues were trimmed, were minced with sterilized forceps. Tissues were digested with 20 mL of 0.2% Collagenase Type 2 (Worthington, Columbus, OH, USA, cat# CLS-2) in Hanks’ Balanced Salt Solution(-) for 45 min at 37 °C mixing with a spin bar. Then the supernatant was collected in the equivalent amount of cooled DMEM/F12 (Gibco, Grand Island, NY, USA, cat# 11330-057) with 10% fetal bovine serum, 0.5% penicillin and streptomycin (Gibco, cat# 15140-122), and 50 μg/mL gentamicin. The process was repeated twice using the fresh digestive agent. Following the filtration with 100 and 40 μm cell strainers and centrifugation at 400× *g* for 5 min, cells were resuspended with growth media: DMEM/F12 containing 20% FBS, supplement mix (Promocell, Heidelberg, Germany, cat# C-39365), 0.5% penicillin and streptomycin, and 50 μg/mL gentamicin. The cells were seeded to regular plastic flasks for the pre-plating to remove fibroblasts from the cell population for 1–2 h. The supernatant containing floating cells was transferred into flasks coated with 0.4% gelatin derived from porcine skin (Sigma-Aldrich, St. Louis, MO, USA, cat# G2500). The primary skeletal muscle cells were passaged three to five times in the growth media, and the batches of the cells were frozen and stored for subsequent experiments to minimize the effect of the heterogeneous cell population between cell batches. Cells were differentiated to myotubes with DMEM/F12 with 2% horse serum heat-inactivated, 1× ITS (Sigma-Aldrich, cat# I3146-5ML), and 0.5% penicillin and streptomycin.

### 4.15. In Vitro PMO Transfection

ASO transfection was performed as previously described [39] with modifications for the primary pig skeletal muscle cells, as shown in Figure S2A,B. In brief, the cells at the density of 2.6 × 10^4^/cm^2^ were seeded in collagen type I-coated culture plates, then cultured in the growth media described above. On day 2 after the seeding, the cells at the density of >95% were differentiated to myotubes using the differentiation media. In 3 days after the differentiation, the spent media were replaced with fresh ones containing unmodified or P7-conjugated PMOs. In 2 or 1 day, the PMOs were removed. Following the culture of 4 or 2 days, the cells were collected for subsequent experiments with mRNA and protein [39].

### 4.16. Exon Skipping Efficiency

The feasibility of exon skipping in DMD pig skeletal muscle cells was tested using PMOs or P7-PMOs with select ASO sequences [63]. RT-PCR was performed as described above. Skipping percentage from the band intensity non-saturated was quantified by ImageJ software (NIH) as follows: exon-skipping efficiency (%) = exons skipped transcript intensity/(native + exon skipped transcript intensity) × 100. Bands of the expected size for the exon-skipped transcript were extracted using a gel extraction kit (Promega). The direct sequencing of the skipped bands was performed with Big Dye Terminator v3.1 (Applied Biosystems). 

## Figures and Tables

**Table 1:** Pig morpholino sequences and their predicted efficiency of skipping pig exon 51 or 53.

Name	Oligo Sequence (5′ to 3′)	Target Exon	mer	Predicted Skip %	Ranking	Distance from Ac
pEx51_Ac0	GTGTCACCAGAGTAACAGTCTGACTAGTAG	51	30	79.7	6	0
pEx51_Ac5	GGGTTGTGTCACCAGAGTAACAGTCTGACT	51	30	89.1	1	5
pEx51_Ac48	ATGGCATTTCTGGTTTGGAGATGGCAGTTT	51	30	37.1	80	48
pEte_Ac65	CTCCAACAGCAAGGAAGATGGCATTTCTGG	51	30	55.4	34	65
pDri_Ac67	GCAAGGAAGATGGCATTTCT	51	20	NA	NA	67
pEx53_Ac9	GTTCCTGGACCTCATCCCACTGACTCTGTA	53	30	88.8	1	9
pEx53_Ac17	CTGAAGGTGTTCCTGGACCTCATCCCACTG	53	30	77.8	7	17
pEx53_Ac18	TCTGAAGGTGTTCCTGGACCTCATCCCACT	53	30	70.0	16	18
pEx53_Ac26	CCTTCTGTTCTGAAGGTGTTCCTGGACCTC	53	30	75.6	10	26
pEx53_Ac30	GTTGCCTTCTGTTCTGAAGGTGTTCCTGGA	53	30	53.4	30	30

Underlines indicate the different bases between pigs and humans. pEte, pig version of eteplirsen; pDri, pig version of drisapersen; NA, not available; Ac, acceptor splice site.

## Data Availability

The data used and/or analyzed during the current study is available from the corresponding author on reasonable request.

## Supplementary Materials

The following are available: https://www.mdpi.com/article/10.3390/ijms222313065/s1.
