# Supplementary material for: A Dystrophin Exon-52 Deleted Miniature Pig Model of Duchenne Muscular Dystrophy and Evaluation of Exon Skipping
Source: Int J Mol Sci. 2021 Dec 2;22(23):13065. doi: 10.3390/ijms222313065 (PMC8657897; doi:10.3390/ijms222313065)
Supplement: Supplementary file 1 [file ijms-22-13065-s001.zip › Supple_Fig_Table_DMDpig_ExonSkip_20211201ye.pdf]

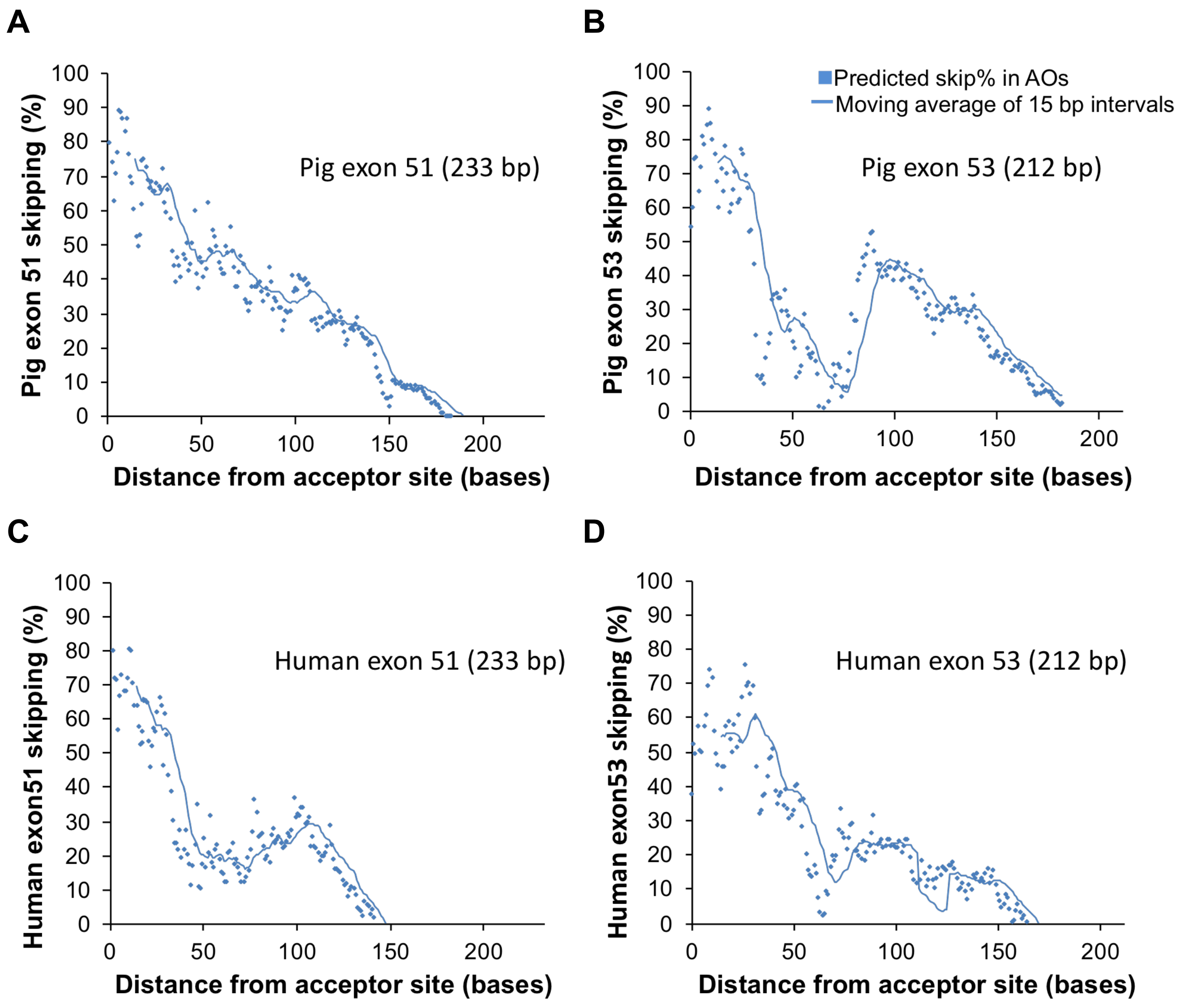

Figure S1. Predictive efficiency of skipping pig *DMD* exon 51 (A) and exon 53 (B) using the in silico tool to design 30-mer PMOs [39]. A similar trend in skipping efficiency between pig and human exons was confirmed. The prediction of human exon 51 and 53 skipping efficiencies (C and D) was visualized for the present study adapted from our previous studies (Echigoya et al., [39] and [38], respectively).

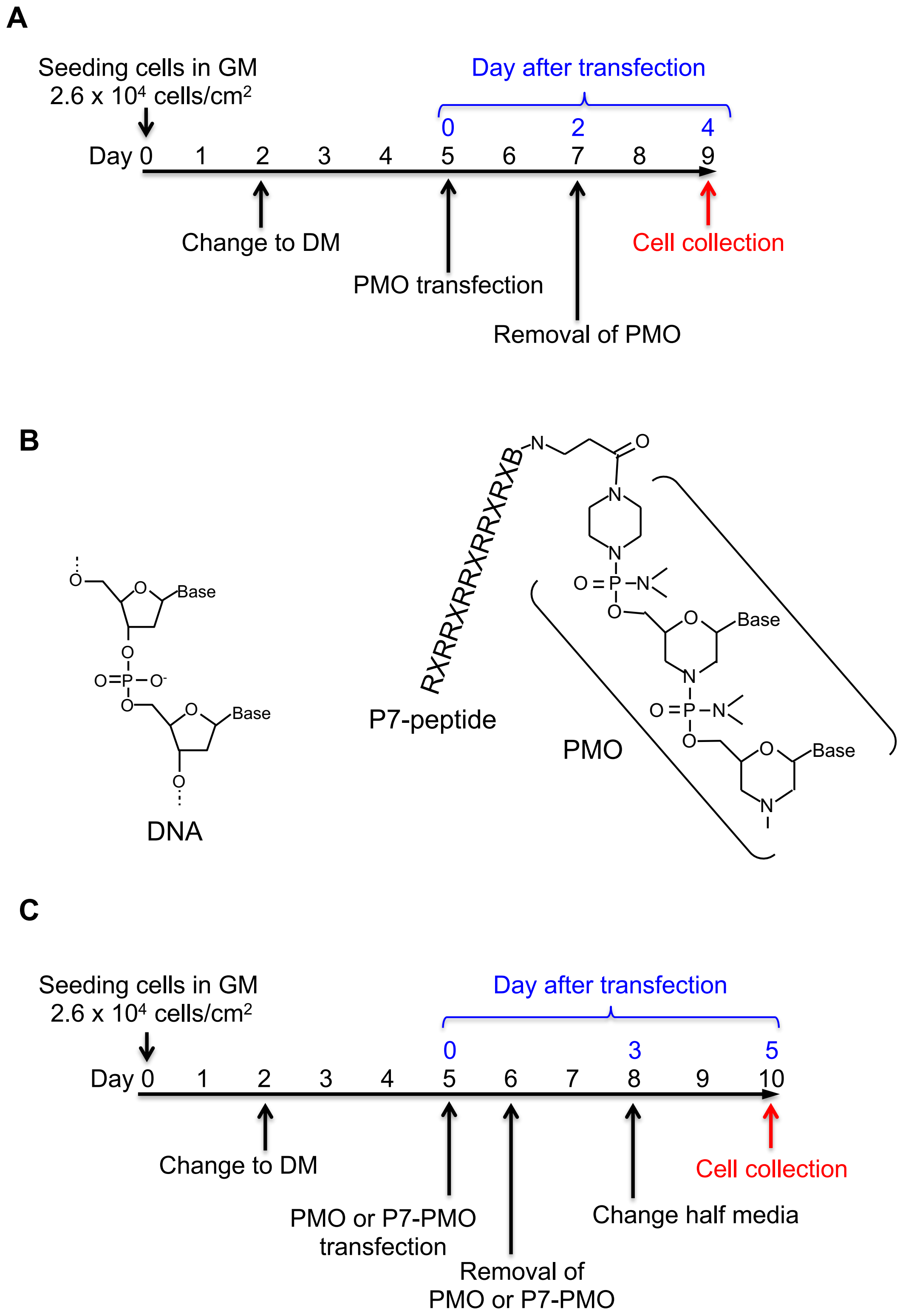

Figure S2. Schematics of transfection conditions of primary DMD pig skeletal muscle cells in the screening test of PMOs at ten-µM (A) and in the comparison of P7-peptide (B)-conjugated and unmodified PMOs (C) as shown in Fig. 6 and 7, respectively. GM, growth media; DM, differentiation media. P7-PMO chemistry (right) compared to DNA chemistry (left): B, β-alanine; X, 6-aminohexanoic acid; R, L-arginine.

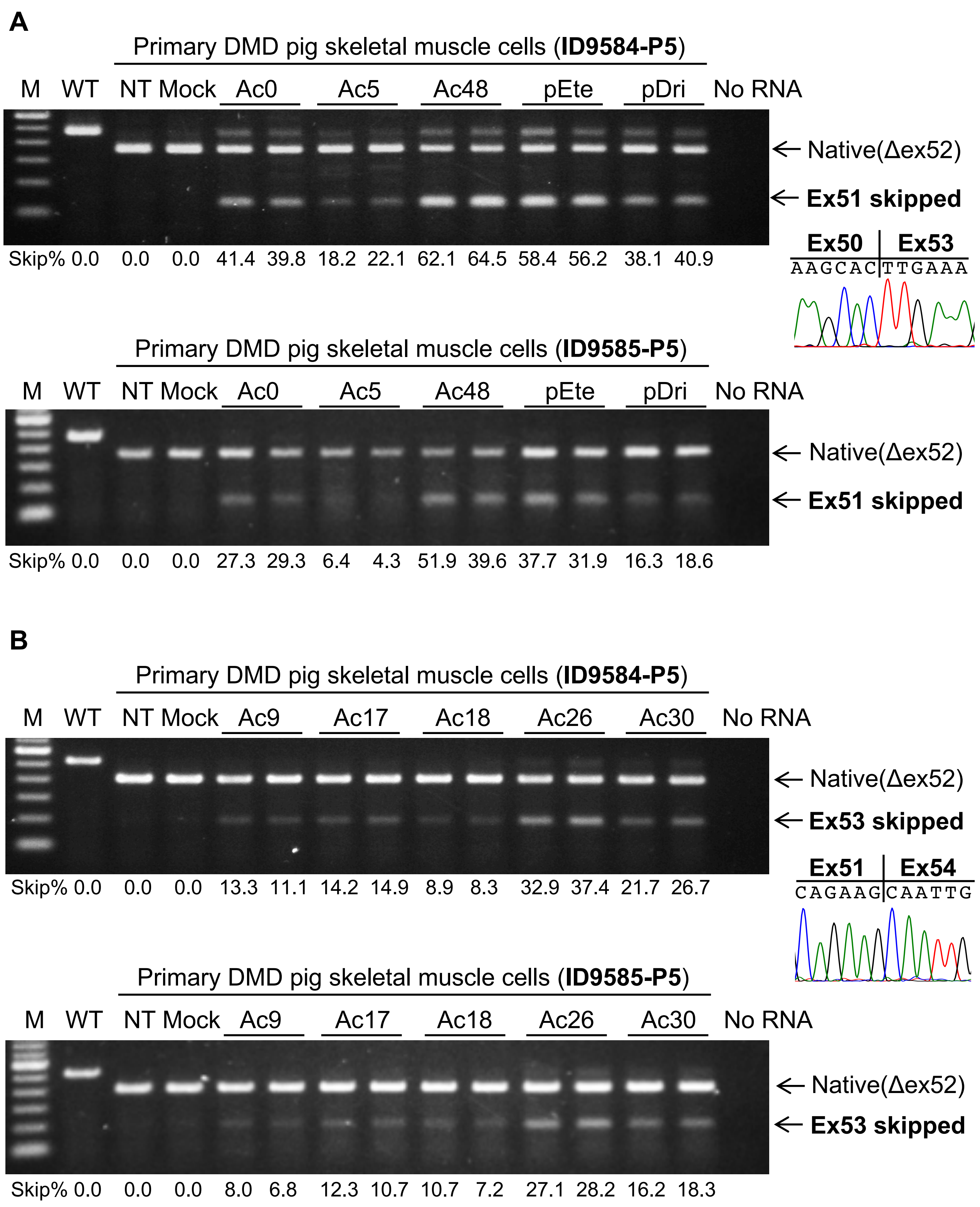

Figure S3. Screening of the designed PMOs to skip exon 51 (A) or exon 53 (B) in the primary skeletal muscle cells derived from two affected male pigs (IDs 9584 and 9585). Ten-μM PMOs were transfected to the DMD pig skeletal muscle cells (Fig. S2A). The percentage of the skipping efficiency was calculated with the following formula: Skip% = skipped band/(native and skipped bands) x 100. The exon junction as represented by colour waves was confirmed by direct sequencing. P5, passage number 5; M, 100 bp marker; WT, wild-type pig-derived primary skeletal muscle cells; NT, non-treated.; Ac, distance from Acceptor slice site.

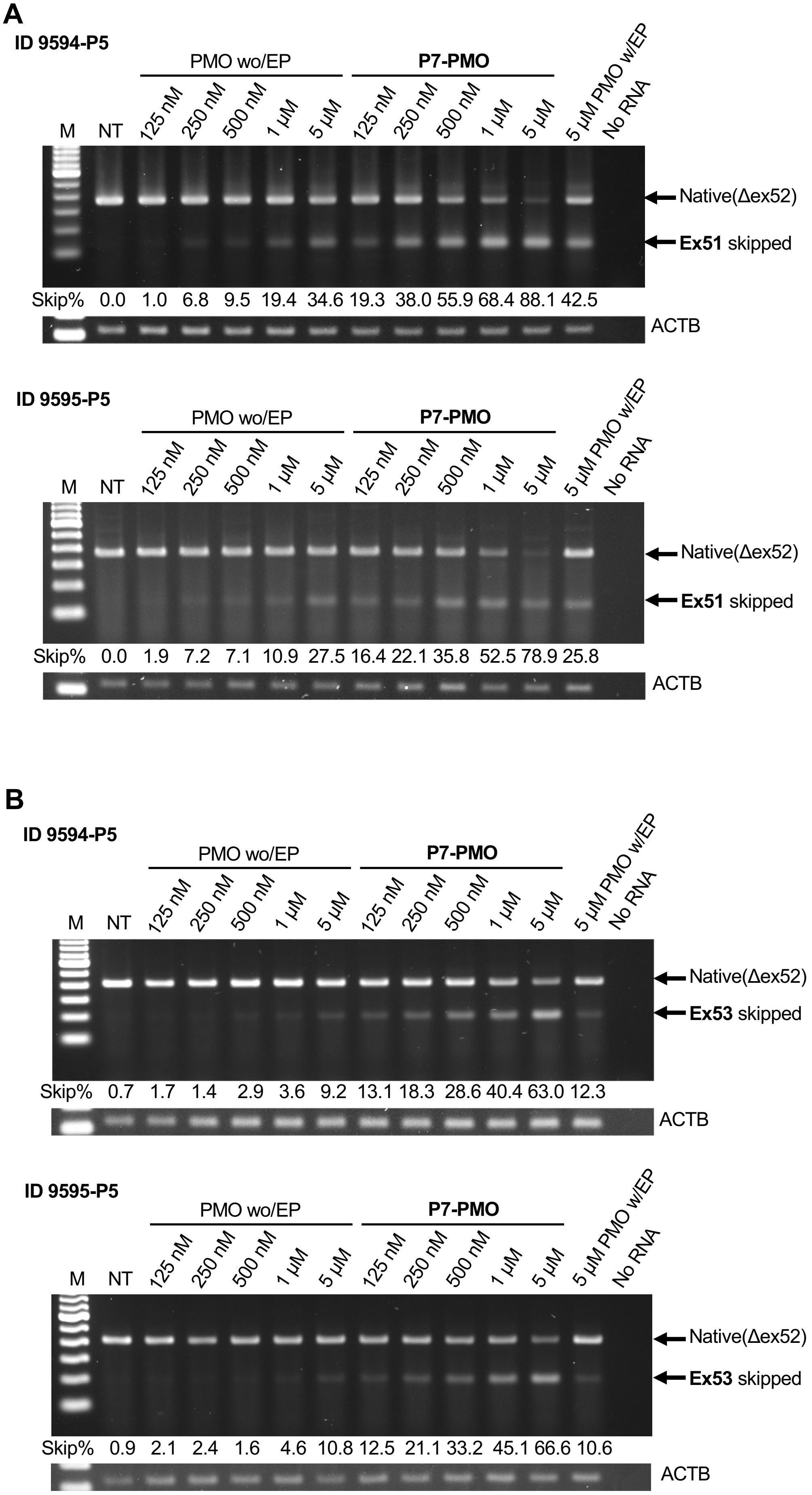

Figure S4. *In vitro* efficacy of P7-peptide-conjugated PMOs composed of Ac48 and Ac26 sequences at skipping exon 51 (A) and exon 53 (B), respectively, in 2 different primary DMD pig skeletal muscle cells with the passage number 5 (P5) (IDs 9584 and 9585). M, 100 bp marker; WT, wild-type pig-derived primary skeletal muscle cells; NT, non-treated; EP, a transfection reagent Endo-Porter peptide.

Table S1. Information of pigs used in this study.

	ID		Age euthanized	Used for
$DMD^{ex52del}$	4 stillborn	Littermates	NA	
	9292		1 day	Histology, CK measurement.
	9293		1 day	Histology, CK measurement.
	9290		5 days	Histology, CK measurement.
	9286		6.5 months	Histology, CK measurement, Growth curve.
	9291		7 months	Histology, Growth curve.
	9584	Littermates	6 days	Primary culture.<br>: Euthanized due to diminished body condition.
	9585		6 days	Primary culture<br>: Euthanized due to diminished body condition.
Wild-type	11380		7 months	Histology, CK measurement, Growth curve.
	11384		8 months	Histology, CK measurement, Growth curve.
	11390		8 months	Histology, CK measurement, Growth curve.
	14425		8 days	Primary culture.
	14426		8 days	Primary culture.

Table S2. PCR primers used in this study.

Purpose	Name	Sequence (5' to 3')
Genotyping	DMD Geno C-F	GATGGATATCTGCAGAATTCG
	DMD Geno E-F	AGCAATCAAGAAGCCAGAACA
	DMD Geno F-F	TGTGGAATGTGTGCGAGGC
	DMD Geno B-R	CATTGCCATTGCTATGGTTCA
RT-PCR	pigDMD_ex50_22-41_Fwd	TCAGAGTGGAAGGCGGTAAC
	pigDMD_ex53_24-44_Rv	AAGGTGTTCCTGGACCTCATC
	pigDMD_ex51_171-191_Fwd	AAATCACAGAGGGTGATGGTG
	pigDMD_ex54_116-136_Rv	AGGAGGCATTGATGTTCTCTG
	pigACTB_Fwd	TCCCTGGAGAAGAGCTACGA
	pigACTB_Rv	GATGCCTGGGTACATGGTG

Table S3. Primary antibodies used in pig muscle tissues or cells.

Antigen	Company	Catalog number	Dilution ratio for pig muscles	
			Western blot	IHC
Human dystrophin rod domain	Leica Microsystems	NCL-DYS1	1/100	1/200
Human dystrophin C-terminal	Leica Microsystems	NCL-DYS2	1/200	1/200
Human dystrophin rod domain	Abcam	ab85302		1/400
Human dystrophin C-terminal	Abcam	ab15277		1/400
Mouse alpha 1-syntrophin	Abcam	ab11187		1/200
Rabbit alpha-sarcoglycan	Leica Microsystems	NCL-a-SARC	1/100	1/10
Human beta-dystroglycan	DSHB	MANDAG2(7D11)		1/5
Human nitric oxide synthase 1	Santa Cruz Biotechnology	sc-8309	1/250	1/5
Human Utrophin	Leica Microsystems	NCL-DRP2		1/5
Chicken alpha Tubulin	Abcam	ab7291	1/15000	
